# Nanostructured, Self-Assembling Peptide K5 Blocks TNF-**α** and PGE_2_ Production by Suppression of the AP-1/p38 Pathway

**DOI:** 10.1155/2012/489810

**Published:** 2012-01-24

**Authors:** Woo Seok Yang, Yung Chul Park, Ji Hye Kim, Hye Ri Kim, Tao Yu, Se Eun Byeon, Larry D. Unsworth, Jaehwi Lee, Jae Youl Cho

**Affiliations:** ^1^Department of Genetic Engineering, Sungkyunkwan University, Suwon 440-746, Republic of Korea; ^2^College of Forest & Environmental Sciences, Kangwon National University, Chuncheon 200-701, Republic of Korea; ^3^Department of Chemical and Materials Engineering, Faculty of Engineering, University of Alberta, Edmonton, AB, Canada T6G 2G6; ^4^College of Pharmacy, Chung-Ang University, Seoul 156-756, Republic of Korea

## Abstract

Nanostructured, self-assembling peptides hold promise for a variety of regenerative medical applications such as 3D cell culture systems, accelerated wound healing, and nerve repair. The aim of this study was to determine whether the self-assembling peptide K5 can be applied as a carrier of anti-inflammatory drugs. First, we examined whether the K5 self-assembling peptide itself can modulate various cellular inflammatory responses. We found that peptide K5 significantly suppressed the release of tumor-necrosis-factor- (TNF-) **α** and prostaglandin E_2_ (PGE_2_) from RAW264.7 cells and peritoneal macrophages stimulated by lipopolysaccharide (LPS). Similarly, there was inhibition of cyclooxygenase- (COX-) 2 mRNA expression assessed by real-time PCR, indicating that the inhibition is at the transcriptional level. In agreement with this finding, peptide K5 suppressed the translocation of the transcription factors activator protein (AP-1) and c-Jun and inhibited upstream inflammatory effectors including mitogen activated protein kinase (MAPK), p38, and mitogen-activated protein kinase kinase 3/6 (MKK 3/6). Whether this peptide exerts its effects via a transmembrane or cytoplasmic receptor is not clear. However, our data strongly suggest that the nanostructured, self-assembling peptide K5 may possess significant anti-inflammatory activity via suppression of the p38/AP-1 pathway.

## 1. Introduction

Inflammation is one of the body innate immune responses and is mainly mediated by macrophages. When viruses or bacteria infect the body, significant cooperation among macrophages, dendritic cells, B cells, and T cells is required. Various inflammatory molecules, such as cytokines (e.g., tumor-necrosis-factor- (TNF-) *α*), chemokines, and mediators (including nitric oxide (NO) and prostaglandin E_2_ (PGE_2_)), are known to play critical roles in managing crosstalk between immune cells in both acute and chronic responses [1, 2]. In this regard, the initial activation of macrophages in inflammatory events could be an important step.

Initiation of the macrophage inflammatory process is triggered by the activation of receptors such as Toll-like receptor (TLR) 4 and TLR3 through the binding of their ligands, such as lipopolysaccharide (LPS) and poly(I:C), respectively [3]. A series of intracellular signaling events follows, managed by the activities of nonreceptor protein tyrosine kinases and mitogen-activated protein kinases (MAPKs), including ERK (extracellular signal-related kinase), p38, and JNK (C-Jun N-terminal kinase), as well as the activation and upregulation of transcription factors (e.g., nuclear-factor- (NF-) *κ*B and activator-protein- (AP) 1) [4, 5]. Eventually, these responses lead macrophages to be transcriptionally activated to express proinflammatory genes encoding such cytokines as tumor-necrosis-factor- (TNF-) *α*, inducible NO synthase (iNOS), and cyclooxygenase- (COX-) 2 [6–9].

Although it is well known that inflammation is a representative defense mechanism in the body, severe inflammatory responses may lead to a number of serious diseases, such as cancer, diabetes, septic shock, rheumatoid arthritis, and atherosclerosis [8, 10–12]. Effective modulation of the acute or chronic inflammatory responses could be used to cure or prevent such diseases; each of the biochemical elements of the inflammatory signaling pathway may be considered as an anti-inflammatory target for new drug development.

Self-assembling peptide nanofiber-based hydrogels with a broad range of biomedical and biotechnological applications have been widely studied [13, 14]. Short peptides with 8 to 16 residues or 2.5 to 5 nm in length have been found to self-assemble into hydrogen-forming nanofibers under the proper conditions, including pH and ionic strength of the medium [15]. Due to their structural merits, such hydrogel systems have been applied to 3D tissue cultures and tissue engineering research systems [16, 17]. An interesting pharmacological application of this nanofiber technology is as a biocompatible drug delivery system controlling the release of small molecules or peptides/proteins [14]. Controlling the release rates of small molecules and peptides/proteins through various hydrogels is a critical aspect of biomaterials science.

Even though self-assembled peptide nanofibers are more advantageous than synthetic drug delivery polymers, it is necessary to test whether the peptide components are immunogenic or inflammation-inductive in the body. Because only a few studies regarding the effects of peptide nanofibers on immunological responses have been reported, we explored the immunoregulatory role of a representative peptide nanofiber (peptide K5) by measuring its inflammation regulation activity *in vitro*.

## 2. Materials and Methods

### 2.1. Materials

Peptide K5 was designed and prepared as previously described [18, 19]. (3-4,5-Dimethylthiazol-2-yl)-2,5-diphenyltetrazolium bromide, a tetrazole (MTT), dextran sulfate sodium (DSS), and lipopolysaccharide (LPS, *E. coli* 0111:B4) were purchased from Sigma Chemical Co. (St. Louis, MO). SB203580 was obtained from Calbiochem (La Jolla, CA). Luciferase constructs containing promoters sensitive to NF-*κ*B and AP-1 were gifts from Professors Chung Hae Young (Pusan National University, Pusan, Republic of Korea) and Man Hee Rhee (Kyungpook National University, Daegu, Republic of Korea). Enzyme immunoassay (EIA) kits and enzyme-linked immunosorbent assay (ELISA) kits for determining PGE_2_ and TNF-*α* were purchased from Amersham (Little Chalfont, Buckinghamshire, UK). Fetal bovine serum and RPMI1640 were obtained from GIBCO (Grand Island, NY). RAW264.7 and HEK293 cells were purchased from the ATCC (Rockville, MD). All other chemicals were of analytical grade and were obtained from Sigma. Phosphospecific or total antibodies against c-Fos, c-Jun, extracellular signal-related kinase (ERK), c-Jun N-terminal kinase (JNK), p38, mitogen-activated protein kinase kinase 3/6 (MKK3/6), TGF-*β*-activated kinase 1 (TAK1), interleukin-1 receptor-associated kinase 1 (IRAK1), inhibitor of *κ*B*α* (I*κ*B*α*), *γ*-tubulin, and *β*-actin were obtained from Cell Signaling (Beverly, MA).

### 2.2. Animals

C57BL/6 male mice (6 to 8 weeks old, 17 to 21 g) were obtained from Daehan Biolink (Chungbuk, Republic of Korea) and housed in plastic cages under conventional conditions. Water and pellet diets (Samyang, Daejeon, Republic of Korea) were available *ad libitum*. Studies were performed in accordance with guidelines established by the Kangwon University Institutional Animal Care and Use Committee.

### 2.3. Preparation of Peritoneal Macrophages

Peritoneal exudates were obtained from C57BL/6 male mice (7 to 8 weeks old, 17 to 21 g) by lavaging four days after intraperitoneal injection of 1 mL of sterile 4% thioglycolate broth (Difco Laboratories, Detroit, MI) as reported previously [20]. After washing with RPMI 1640 medium containing 2% FBS, peritoneal macrophages (1 × 10^6^ cells/mL) were plated in 100 mm tissue culture dishes for 4 h at 37°C in a 5% CO_2_ humidified atmosphere.

### 2.4. Cell Culture

Peritoneal macrophages and cell lines (RAW264.7 and HEK293 cells) were cultured with RPMI1640 medium supplemented with 10% heat-inactivated fetal bovine serum (Gibco, Grand Island, NY), glutamine, and antibiotics (penicillin and streptomycin) at 37°C under 5% CO_2_. For each experiment, cells were detached with a cell scraper. At the cell density used for experiments (2 × 10^6^ cells/mL), the proportion of dead cells was less than 1% as measured by Trypan blue dye exclusion.

### 2.5. NO, PGE_2_, and TNF-*α* Production

After preincubation of RAW264.7 cells or peritoneal macrophages (1 × 10^6^  cells/mL) for 18 h, cells were pretreated with Peptide K5 (0 to 400 *μ*g/mL) for 30 min and were further incubated with LPS (1 *μ*g/mL) for 24 h. The inhibitory effect of peptide K5 on NO, PGE_2_, and TNF-*α* production was determined by analyzing NO, PGE_2_, and TNF-*α* levels with the Griess reagent and enzyme linked immunosorbent assay (ELISA) kits as described previously [21, 22].

### 2.6. Cell Viability Test

After preincubation of RAW264.7 cells (1 × 10^6^ cells/mL) for 18 h, the peptide K5 (0 to 100 *μ*g/mL) was added to the cells and incubated for 24 h. The cytotoxic effect of the peptide K5 was then evaluated by a conventional MTT assay, as reported previously [23, 24]. At 3 h prior to culture termination, 10 *μ*L of MTT solution (10 mg/mL in phosphate-buffered saline, pH 7.4) was added to each well, and the cells were continuously cultured until termination of the experiment. The incubation was halted by the addition of 15% sodium dodecyl sulfate into each well, thus solubilizing the formazan [25]. The absorbance at 570 nm (OD_570–630_) was measured using a Spectramax 250 microplate reader. 

### 2.7. mRNA Analysis by Real-Time Polymerase Chain Reactions (RT-PCR)

To determine cytokine mRNA expression levels, total RNA was isolated from LPS-treated RAW264.7 cells with the TRIzol Reagent (Gibco BRL) according to the manufacturer's instructions. Total RNA was stored at −70°C until use. Quantification of mRNA was also performed using real-time RT-PCR with SYBR Premix Ex Taq (Takara, Japan) and a real-time thermal cycler (Bio-Rad, Hercules, CA) as reported previously [26, 27]. The results were expressed as a ratio of optical density to GAPDH. The primers (Bioneer, Daejeon, Republic of Korea) used are indicated in Table 1.

### 2.8. Luciferase Reporter Gene Activity Assay

HEK293 cells (1 × 10^6^ cells/mL) were transfected with 1 *μ*g of plasmids containing NF-*κ*B-Luc or AP-1-Luc along with *β*-galactosidase using the calcium phosphate method in a 12-well plate according to the manufacturer's protocol [28]. The cells were used for experiments 48 h after transfection. Luciferase assays were performed using the Luciferase Assay System (Promega) as reported previously [29]. 

### 2.9. Preparation of Total Lysates and Nuclear Extracts and Immunoblotting

Preparation of total lysates and nuclear extracts from LPS-treated RAW264.7 cells pretreated with peptide K5 was performed using a previously published method [30]. Immunoblot analysis of levels of phosphorylated or total transcription factor (AP-1(c-Fos, c-Jun), p-FRA-1), Lamin A/C, MAPK (ERK, p38, and JNK), MKK 3/6, TAK-1, IRAK-1, I*κ*B*α*, and *β*-actin was performed according to published methods [31, 32].

### 2.10. Statistical Analysis

**Figure 1:**
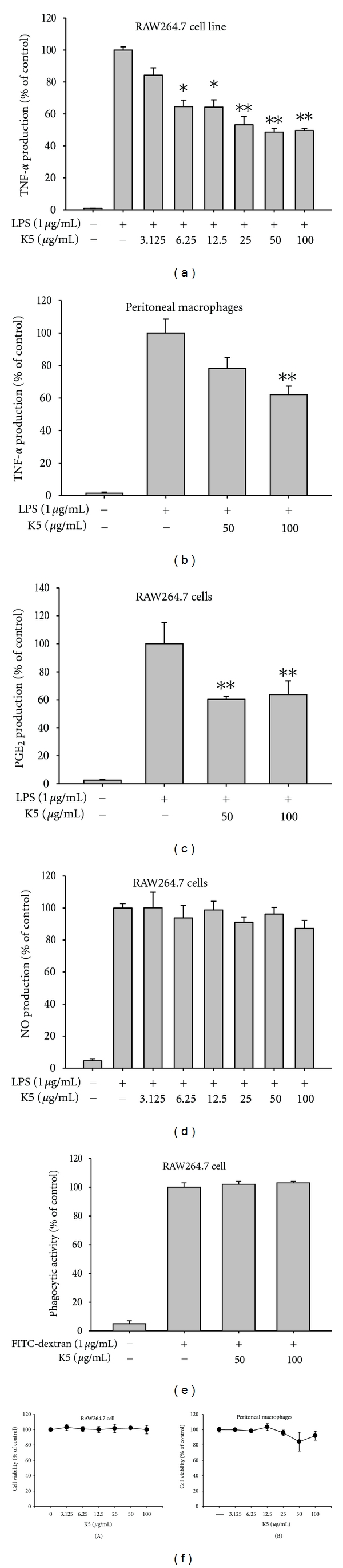
Effect of peptide K5 on the production of inflammatory mediators. (a), (b), (c), and (d) Levels of NO, PGE_2_, and TNF-*α* were determined by Griess assay, EIA, and ELISA from culture supernatants of RAW264.7 cells or peritoneal macrophages treated with peptide K5 (0 to 100 *μ*g/mL) and LPS (1 *μ*g/mL) for 6 (TNF-*α*) or 24 (NO and PGE_2_) h. (e) Phagocytic uptake levels of FITC-dextran were determined by flow cytometric analysis using RAW264.7 cells treated with peptide K5 (0 to 100 *μ*g/mL) and FITC-dextran (1 *μ*g/mL) for 6 (TNF-*α*) or 24 (NO and PGE_2_) h. (e) Cell viability of RAW264.7 cells and peritoneal macrophages was determined by MTT assay. *P < 0.05 and **P < 0.01 compared to control.

**Figure 2:**
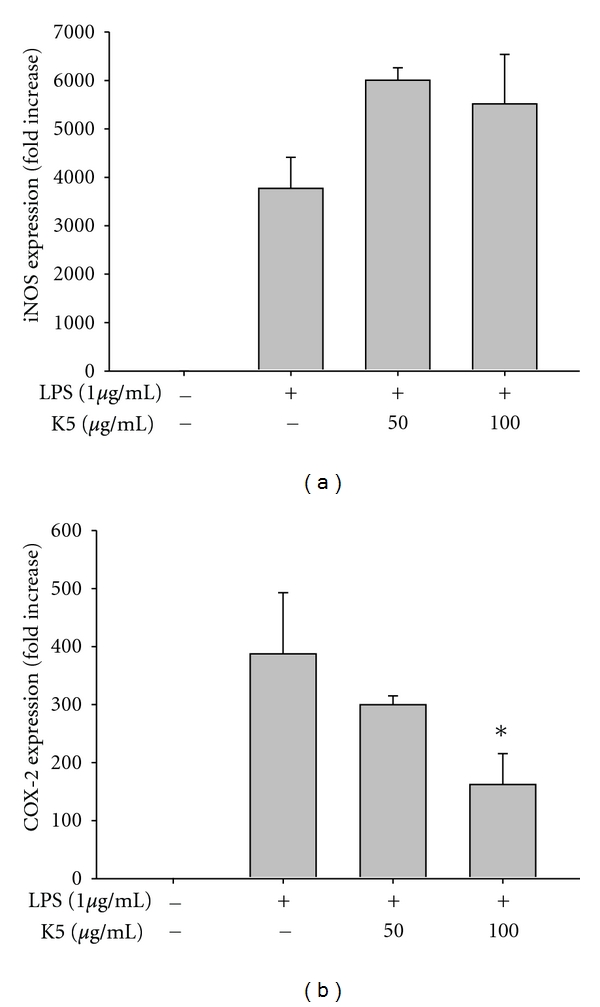
Effect of peptide K5 on the mRNA expression of proinflammatory genes. (a) and (b) The mRNA levels of iNOS and COX-2 were determined by real-time PCR. *P < 0.05 compared with controls.

Data (Figures 1, 2, and 4), expressed as mean ± standard deviation (SD), were calculated from one (*n* = 6) of two independent experiments. Other data are representative of three different experiments with similar results. For statistical comparisons, results were analyzed using analysis of variance/Scheffe's post hoc test, and the Kruskal-Wallis/Mann-Whitney test. All *P* values <0.05 were considered statistically significant. All statistical tests were carried out using the computer program SPSS (SPSS Inc., Chicago, IL).

## 3. Results and Discussion

Peptide K5 is a representative peptide known to be self-assembled and to form nanofibers that can be used as 3D scaffolds for tissue engineering or drug delivery [14, 17]. Thus far, no one has tested whether this peptide itself can induce an immunological response, but it is important for us to determine whether this nanofiber is immunogenic prior to using it as a drug delivery system. Therefore, in this study, the regulatory activity of peptide K5 on macrophage-mediated inflammatory responses was examined under LPS treatment conditions.

Our data suggest that peptide K5 can act as a therapeutic molecule with anti-inflammatory properties. Thus, K5 suppressed TNF-*α* production in a concentration-dependent manner in both in RAW264.7 cells (Figure 1(a)) and in bone marrow-derived macrophages stimulated by LPS (Figure 1(b)). The peptide also showed significant inhibition of PGE_2_ production in LPS-treated RAW264.7 cells (Figure 1(c)). However, peptide K5 did not suppress either NO release from LPS-treated RAW264.7 cells (Figure 1(d)) or phagocytic uptake of FITC-dextran by RAW264.7 cells (Figure 1(e)), indicating that not all macrophage activation pathways are blocked by the peptide. Because K5 did not induce cytotoxicity at up to 100 *μ*g/mL under the conditions studied (Figure 1(f)), all inhibitory activities seem to be due to a specific pharmacological action. The suppressive activities were much stronger than or comparable to those of previously reported peptides such as an immunomodulatory peptide (CP), human *β*-defensin 3, HBD-2, and enantiomeric 9-mer peptide [33, 34]. Considering these points, it is reasonable that peptide K5 could be used as an anti-inflammatory therapeutic.

**Figure 3:**
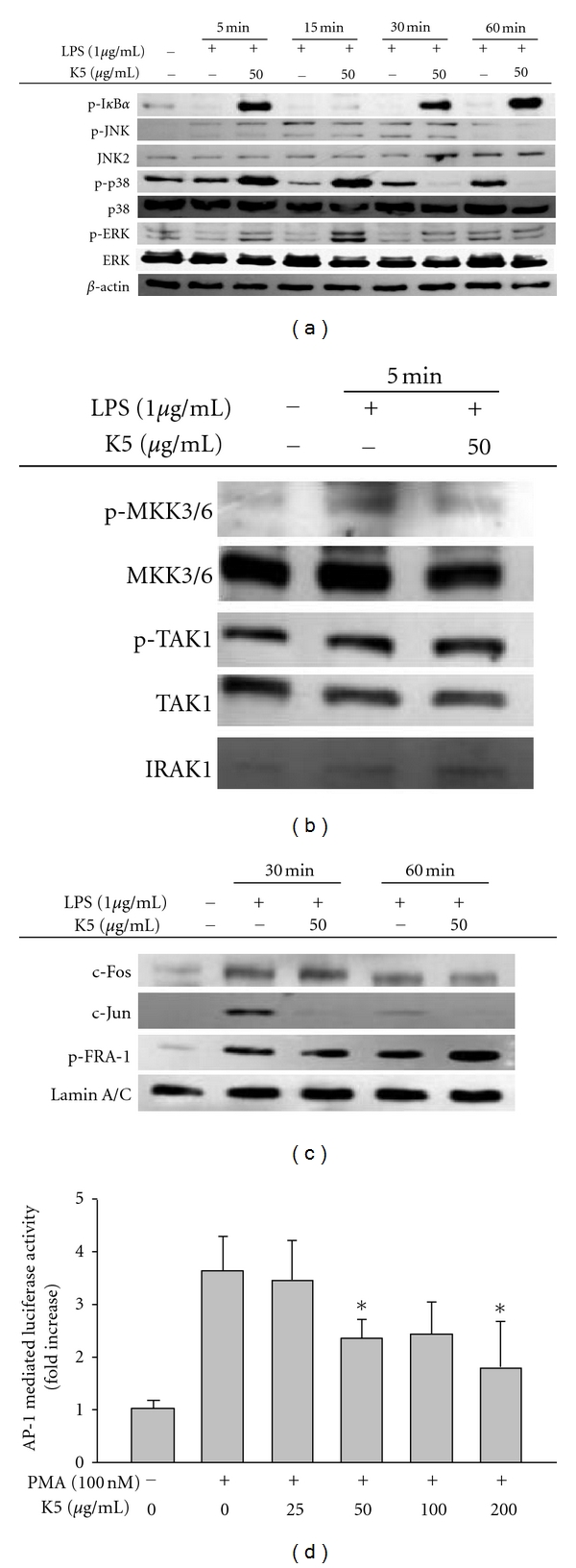
Effect of peptide K5 on the activation of signaling events upstream of AP-1 activation. (a) and (b) Phosphoprotein or total protein levels of I*κ*B*α*, ERK, p38, JNK, MKK3/6, TAK1, IRAK1, and *β*-actin from cell lysates were determined by Western blot analysis using phosphospecific or total protein antibodies. (c) Levels of members of the AP-1 family (p-Fra-1, c-Jun, and c-Fos) in the nuclear fraction were determined by Western blot analysis using antibodies against total protein. (d) HEK293 cells cotransfected with plasmid constructs NF-*κ*B-Luc, or AP-1-Luc (1 *μ*g/mL each) and *β*-gal (as a transfection control) were treated with SB203580 (25 *μ*M) in the presence or absence of TNF-*α* (20 ng/mL) for NF-*κ*B activation or PMA (10 ng/mL) for AP-1 activation. Luciferase activity was measured using a luminometer. *P < 0.05 compared with controls.

Because drug development requires understanding the molecular mechanism of a drug pharmacological action, we explored the inhibitory mechanism of peptide K5. To determine whether peptide K5 is able to suppress LPS-induced inflammatory responses at the transcriptional or translational levels, the mRNA levels of the inflammatory genes iNOS and COX-2 induced by LPS were measured by real-time PCR. Peptide K5 only blocked mRNA expression of COX-2 up to 60% at 100 *μ*g/mL (Figure 2), implying that the inhibition of PGE_2_ production could be due to the blockade of transcriptional activation mediated by inflammatory transcription factors, such as NF-*κ*B and AP-1 [35]. To determine which transcription factors are targeted by peptide K5, nuclear proteins were analyzed. Interestingly, at 30 to 60 min, peptide K5 markedly diminished nuclear levels of c-Jun, while Fra-1 and c-Fos levels were not altered (Figure 3(a)), indicating that, among the different AP-1 family proteins, c-Jun could be a transcriptional target of peptide K5. To identify the molecular target of peptide K5, signaling events upstream of AP-1 translocation were continuously evaluated. Intriguingly, at 30 to 60 min, peptide K5 markedly suppressed the phosphorylation of p38, a critical enzyme for c-Jun translocation (Figure 3(b)), although this peptide remarkably enhanced p38 phosphorylation. Meanwhile, because there was no inhibition of I*κ*B*α* phosphorylation, and rather it has been enhanced at 5, 30, and 60 min (Figure 3(b)), we expected that NF-*κ*B, a major transcription factor activated under inflammatory conditions, would not be blocked by peptide K5. Indeed, the peptide did not suppress NF-*κ*B translocation (data not shown). So far, we could not understand why the phosphorylation of I*κ*B*α* was enhanced by the treatment of peptide K5. Whether this peptide is able to stimulate the activity of upstream signaling enzymes such as Akt and I*κ*B*α* kinase (IKK) for this event or increase the protein level of I*κ*B*α* by suppression of its cleavage pathway could be considered to be tested in the following experiments.

To determine whether peptide K5 can block events upstream of p38, the phosphorylation patterns of several upstream enzymes were investigated. Peptide K5 inhibited the phosphorylation of MKK3/6, an upstream kinase of p38 [36], at 5 min, while the activation of its upstream enzymes, TAK1 and IRAK1, as assessed by measuring phospho-TAK1 and a degraded form of IRAK1 [37], was not diminished (Figure 3(c)). Peptide K5 also showed an inhibitory pattern of luciferase activity mediated by AP-1 (Figure 3(d)), as assessed by a reporter gene assay with AP-1 promoter. Therefore, our data strongly suggest that the molecular target of peptide K5 seems to be a complex of AP-1 (c-Jun), p38, MKK3/6 kinase, and TAK1 in LPS signaling. However, whether this peptide can block TAK1 enzymatic activity either directly or indirectly is not yet clear. Detailed studies using purified TAK1 will be conducted to address this issue.

**Figure 4:**
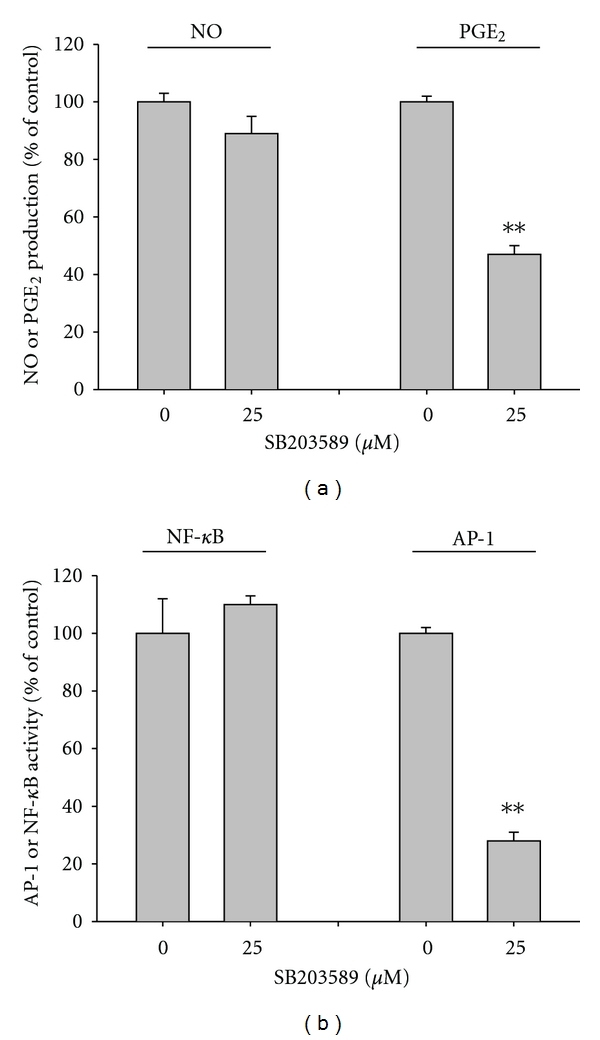
Effect of SB203580 on the production of NO and PGE_2_ and activation of AP-1. (a) Levels of NO and PGE_2_ were determined by Griess assay and EIA from culture supernatants of RAW264.7 cells treated with SB203580 (25 *μ*M) and LPS (1 *μ*g/mL) for 24 (NO and PGE_2_) h. (a) HEK293 cells cotransfected with plasmid constructs NF-*κ*B-Luc or AP-1-Luc (1 *μ*g/mL each) and *β*-gal (as a transfection control) were treated with SB203580 (25 *μ*M) in the presence or absence of TNF-*α* (20 ng/mL) for NF-*κ*B activation or PMA (10 ng/mL) for AP-1 activation. Luciferase activity was measured using a luminometer. **P < 0.01 compared with controls.

The functional significance of the p38/MKK3/6/TAK1 pathway for AP-1 activation and inflammatory responses has been well defined [36]. To confirm its role under our experimental conditions, a selective inhibitor of p38, SB203580, was employed and tested under the same conditions. Neither K5 nor the p38 inhibitor blocked NO production (Figure 4(b)). In contrast, SB203580 markedly suppressed both PGE_2_ (Figure 4(b)) and TNF-*α* production (data not shown), suggesting that the p38 pathway may be a critical component in regulating the release of PGE_2_ and TNF-*α* in LPS-treated macrophages.

In general, it is known that proteins and peptides are not able to penetrate the cell membrane due to their sizes and charges. Small hydrophobic molecules exhibiting anti-inflammatory activities, however, are membrane-permeable compounds and are expected to easily modulate cytosolic target enzymes. In this regard, the possibility that peptide K5 can be transported into the cytoplasm by a specific transporter seems plausible. Several receptors, such as PEPT1, OATP1A2, OATP1B1, OATP1B3, and OATP2B1, have been identified as transporters of peptide molecules [38, 39]. Because some of these receptors, including PEPT1, have been reported to be involved in inflammatory responses, it is necessary to test whether these transporters and the relevant signaling pathways are critical for the pharmacology of peptide K5. Further studies will be conducted to address these points.

Unlike small-molecule drugs, the therapeutic use of peptide-based drugs is hindered by cost, stability, pharmacokinetics, and obscure targeting. Nonetheless, some peptides such as GLP-1 analogs, extracellular matrix protein-derived peptides such as acetylated Pro-Gly-Pro (Ac-PGP) [40], and integrin-binding N-terminal peptide [41] have been reported to have anticancer, antiseptic shock, and antitumorigenic activities and were subsequently developed as promising peptide drugs. Indeed, some peptide-type drugs have already been used as glycopeptide-type antibiotics (e.g., vancomycin) [42]. However, compared with small-molecule drugs having subnanomolar potency, it is difficult to develop a low-potency peptide into a drug. Recent studies have focused on developing special drug delivery apparatuses using peptides [14]. Peptide K5 is a candidate peptide for drug delivery; it is a self-assembling nanopeptide forming a hydrogel and is known to be generally nonimmunogenic, nonthrombogenic, and applicable to such noninvasive therapies as void filling [14, 18, 19]. Through these properties, self-assembling peptides have been nominated as scaffolds for 3D cell culturing systems, regenerative medicine applications, and drug delivery applications [43, 44]. Although such applicable features are sufficient to warrant use of these peptides, the anti-inflammatory property of peptide K5 could provide an additive benefit when used as an anti-inflammatory drug delivery system. In particular, because this peptide has been shown to block the p38/AP-1 pathway in the LPS/TLR4 response, drugs with inhibitory activities on the activation of NF-*κ*B or IRF-3, which affect the production of NO and IFN-*β*, will help to synergize total anti-inflammatory responses. This hypothesis will be evaluated using several *in vivo* inflammatory models, such as DSS-induced colitis and collagen-type-II-generated arthritis.

**Figure 5:**
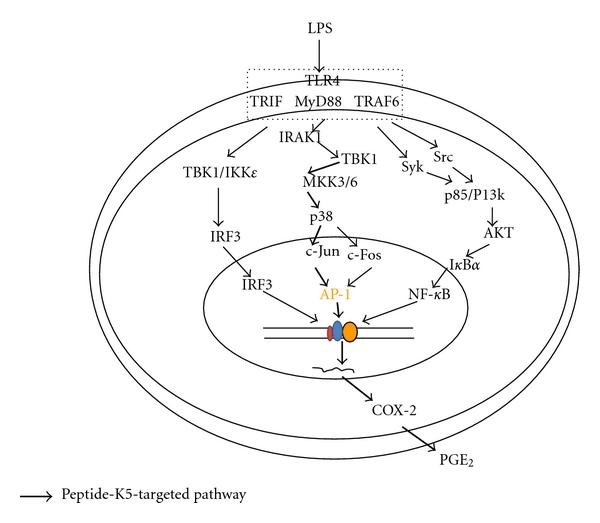
Putative inhibitory pathway of LPS-activated inflammatory signaling responses by peptide K5.

In summary, we have demonstrated that peptide K5 can strongly block macrophage-mediated inflammatory responses, such as the production of TNF-*α* and PGE_2_, during the activation of TLR4. In particular, peptide K5 selectively diminishes the activation of AP-1 by suppression of upstream signaling enzymes, such as MKK3/6 and TAK1 (Figure 5). Considering that this peptide is a self-assembling drug delivery carrier, it is possible that peptide K5 could be developed as a carrier of NF-*κ*B inhibitory drugs and act synergistically to combat inflammation. To investigate this possibility, we plan to conduct additional *in vivo* efficacy tests using models of chronic inflammatory disorders (e.g., colitis and arthritis).

## Figures and Tables

**Table 1:** Sequences of primers used in real-time PCR analysis.

Gene		Primer sequences
iNOS	F	5′-CCCTTCCGAAGTTTCTGGCAGCAGC-3′
	R	5′-GGCTGTCAGAGCCTCGTGGCTTTGG-3′
COX-2	F	5′-CACTACATCCTGACCCACTT-3′
	R	5′-ATGCTCCTGCTTGAGTATGT-3′
GAPDH	F	5′-CACTCACGGCAAATTCAACGGCAC-3′
	R	5′-GACTCCACGACATACTCAGCAC-3′
